# Understanding trial informativeness in digital mental health: perspectives from researchers and lived experience experts

**DOI:** 10.1186/s13063-026-09610-w

**Published:** 2026-03-18

**Authors:** Alexia Jeayes, Camilla Babbage, Kirsty Sprange, Charlotte L. Hall

**Affiliations:** 1 Mental Health & Clinical Neurosciences, MindTech Medtech Co-operative, School of Medicine, University of Nottingham, Nottingham, UK; 2 Nottingham Clinical Trials Unit, School of Medicine, University of Nottingham, Nottingham, UK; 3 NIHR Nottingham Biomedical Research Centre School of Medicine, University of Nottingham, Nottingham, UK

**Keywords:** Mental health, Digital therapeutics, Young people, Children, UK, Trials, Informativeness

## Abstract

**Background:**

Randomised controlled trials (RCTs) are often considered the gold standard in research design to determine clinical and cost-effectiveness, and yet the majority are not considered “*informative”* or otherwise useful for decision-makers in research, clinical practice, and policy. What constitutes an informative trial of digital mental health interventions (DMHIs) for children and young people (CYP) remains unclear. By investigating the perspectives of researchers and young people, this study highlights key components that enhance the relevance, rigour, and impact of youth DMHI trials.

**Methods:**

This multi-method qualitative study, guided by a pragmatist approach, explored the perspectives of researchers, with expertise in DMHIs, and young people, with lived experience of mental health conditions and DMHI use, on what is an informative DMHI trial for CYP (RQ1) and what contributes to informativeness (RQ2). Semi-structured interviews with researchers were analysed using reflexive thematic analysis, whilst the content of participatory group workshops with young people were summarised in relation to researcher-generated themes.

**Results:**

Seven researchers and six young people participated. Four themes and 10 sub-themes were generated for what an informative trial is: 1) Addresses questions important to partners; 2) Feasible to conduct; 3) Trustworthy and credible, and 4) Accessible and safe. Three themes were generated as contributors to informativeness, including 5) Partner involvement; 6) Researcher and site capacity, and 7) The wider context. Both groups recognised the importance of informative trials, but researchers emphasised methodological rigour and logistics, whereas young people focused on broader concerns such as the impact of power dynamics.

**Conclusions:**

This study highlights core components of informativeness in DMHI trials for CYP and demonstrates the value of involving both academic and non-academic contributors in shaping informativeness.

**Supplementary Information:**

The online version contains supplementary material available at 10.1186/s13063-026-09610-w.

## Background

Clinical trials play a crucial role in advancing medical science and improving healthcare. Randomised controlled trials (RCTs) are often considered the "gold standard" for evaluating effectiveness. If conducted with rigour, they have the potential to minimise bias and establish cause-and-effect relationships, making them essential for building evidence-based practice [1]. However, reports suggest that many RCTs are not conducted in this way, with 55–62% having a high risk of bias [2, 3], and only 26.4% of trials having all the components considered to be useful for clinical practice [4].

Informativeness refers to the extent to which an RCT is designed, conducted, and reported in a way that generates reliable and meaningful evidence to guide future clinical practice, research, and policy decisions [5, 6]. According to Zarin and colleagues [5], a clinical trial is considered *informative* when it possesses five key attributes. First, the trial must address a research question of substantive importance, with the potential to influence scientific understanding, clinical practice, or policy. Second, its design should be methodologically sound and capable of generating meaningful evidence relevant to the stated hypothesis. Third, the trial must be feasible, with realistic expectations for recruitment. Fourth, it should be conducted and analysed with scientific integrity, adhering closely to the pre-specified protocol. Finally, mechanisms must be in place to ensure the timely, complete, and accurate reporting of results.

In addition to Zarin, several approaches are proposed to enhance trial informativeness. A scoping review identified 27 global best practices, including pragmatic trial designs, partner involvement, and improved reporting standards [7]. Taylore and Kowalkowski [8] emphasised implementation-guided pilot studies, while Dolley and colleagues [9] proposed a maturity model for improving scientific review. O'Dea et al. [10] developed digital mental health intervention (DMHI) trial indicators through a Delphi design.

A key challenge with the practices highlighted above is knowing which to prioritise when developing, delivering and evaluating research, particularly in DMHI trials for children and young people (CYP), where currently no specific guidelines exist. Young people differ fundamentally from adults in several ways, not limited to the impact their developmental trajectories can have on their responses to interventions at a given time [11]. The need to engage collaborators relevant to CYP has been highlighted by previous work showing that different partners prioritise different components of informativeness [10]. Lived experience (LE) refers to the personal knowledge that individuals gain from directly experiencing conditions and services. To our knowledge, no study has directly included the perspectives of LE young people. Involving CYP is essential to ensure that digital mental health trials are genuinely patient-centred and responsive to their needs [12, 13].

This paper investigates two research questions (RQs) in the context of DMHIs for CYP: RQ1, what is an informative trial, and RQ2, what contributes to informativeness. By integrating perspectives from both academic researchers and young people with LE, we seek to identify the key components that enhance the relevance, rigour, and impact of trials in this field to inform the design of more meaningful and effective future trials.

## Methodology

**Fig. 1:**
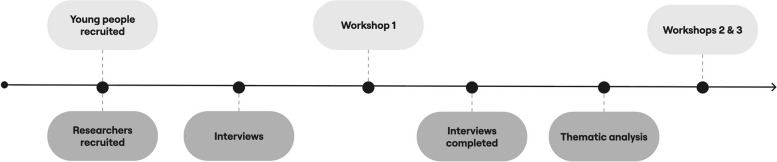
Flow diagram of methodological processes

This study employed a qualitative multi-method design, guided by a pragmatist approach [14, 15], involving one-to-one semi-structured interviews with research experts and group workshops with LE experts to gather diverse perspectives on what makes DMHI trials for CYP informative (see Fig. 1 for methodological processes). Reporting followed the COREQ guidelines [16].

The study was approved by the Research Ethics Committee at the University of Nottingham, Faculty of Medicine and Health Sciences (FMHS 72–0125) on 20th February 2025.

### Recruitment methods

Researchers with experience in conducting DMHI trials for CYP were purposively and snowball-sampled through academic papers, trial registers, public reports, academic networks, and word-of-mouth. Purposive sampling prioritised individual characteristics that were identifiable from the selected recruitment sources, including diverse clinical or academic backgrounds, organisation affiliation, and involvement in DMHI research to improve the richness and credibility of our findings. Personalised invitations were emailed to potential participants, who, upon expressing interest, received a participant information sheet and consent form. All researcher participants provided written informed consent digitally before participation.

#### Lived experience experts

Young people aged 12–25 years with LE of mental health conditions and/or prior use of DMHIs were invited to participate in workshops by convenience sampling. Recruitment was conducted via email newsletter through a third-sector organisation, called the McPin Foundation [17], which aims to facilitate the involvement of LE young people and adults in research and evaluation. Those expressing an interest were invited to a one-to-one meeting with a researcher to learn about the aims of the project and confirm eligibility based on lived experience of mental health conditions and/or prior DMHI use. Although informed consent is not required for public and patient involvement (PPI) activities, all members signed retrospective agreement permitting the inclusion of their contributions in this paper. Their involvement has been reported following the GRIPP2 short form checklist ([18, 19] see appendix).

Demographic data were collected from both young people and researchers to assess the diversity and inclusivity of the sample. All were offered acknowledgement in research outputs and monetary compensation for taking part.

### Data collection methods

#### Semi-structured interviews

Individual interviews with researchers followed a semi-structured guide (see appendix C). The guide was based on the academic literature of informativeness; however, questions were adapted based on the content of each interview. Interviews were conducted and recorded online via Microsoft Teams. These took place between February 2025 – May 2025, lasted approximately 30 min and were conducted by the first author (AJ).

Three 60-min workshops with LE experts were conducted online via Microsoft Teams, using a flexible, participatory approach to encourage open discussion and idea generation [20]. These were designed as a collaborative process to contextualise, refine, and challenge themes emerging from researcher interviews, rather than as a standalone qualitative data collection method. Consistent with a pragmatist and co-production ethos, the workshops were adaptive in structure and positioned LE contributors as collaborators in sense-making rather than research participants. The first workshop took place during interview data collection and focused on broadly exploring the concept of informativeness, while the subsequent workshops delved into interview-generated themes for more in-depth discussions. These workshops took place between April 2025 – July 2025, using a combination of prompted discussion, reflective exercises, and group sense-checking of emerging themes, with outputs documented contemporaneously. These were not audio or video recorded; two facilitators (AJ, CB, SO) documented all discussions and ideas in real time using Miro, a digital collaborative whiteboard platform designed to support collective planning and visualisation [21]. Individual comments on the Miro board were anonymous to encourage open participation. Individuals who could not attend a workshop were invited to participate offline.

### Data analysis methods

#### Semi-structured interviews

All interview recordings and verbatim transcripts were generated from Microsoft Teams. Transcripts were reviewed for accuracy, corrected, and anonymised. Researchers were assigned numerical identifiers and referred to as Researchers (R). Original transcripts and videos containing sensitive information were destroyed after data analysis.

Semi-structured interviews were analysed using deductive and inductive reflexive thematic analysis, involving six recursive stages [22–24]. Deductive codes were generated from the existing literature on informativeness [5], which were discussed and refined by the research team before being applied to all transcripts. However, considering the lack of clarity regarding components of informativeness relevant to this context, inductive codes were also developed.

The researcher (AJ) conducted thematic analysis of anonymised transcripts, initially engaging with the data through listening to interviews, reading transcripts and taking notes to identify early insights into participants’ interpretations of informativeness. Transcripts were imported into NVivo 15 [25] for line-by-line coding, beginning with deductive, then inductive coding. Codes were assigned to relevant text segments and grouped into preliminary themes based on emerging patterns. Visual maps were created using Miro to support theme development. Themes were iteratively reviewed, refined, and defined in relation to the research question, with illustrative quotes selected to support interpretation. The researcher met biweekly with all authors to discuss and refine themes, ensuring collaborative input throughout the process.

#### Participatory group workshops

LE workshops were conducted as part of a collaborative process prior to and after the thematic analysis of researcher interviews. Given the participatory, anonymous, and adaptive nature of the workshops, they were not intended to be replicable in a procedural sense but transparently reported to enable appraisal of how LE perspectives informed analytic interpretation. LE experts reviewed emerging themes, offering reflections, additional perspectives, and contextual insights. Their input was used to enrich the findings but was not subjected to formal qualitative analysis, as the group participated as collaborators rather than research participants. Due to the anonymous nature of their contributions, we could not decipher what LE expert said versus what was written by a researcher who paraphrased any verbal discussion. Their contributions are therefore described in paraphrased form, in conjunction with insights generated from research expert interviews.

### Reflexivity

To enhance transparency and in recognising the researcher’s inevitable positionality when shaping data analysis, the primary researcher journaled within 24 h of each interview and during data analyses. These practices enabled an adaptive approach, allowing for more meaningful engagement with participants while also enhancing awareness of the impact the researcher had on analysis. For example, reflexivity in reviewing interview data allowed the researcher to identify missed opportunities for exploring participants’ perspectives more deeply. Reflexivity allowed the researcher to adapt the approach in subsequent interviews, prompting them to revisit topics where more understanding of perspectives was needed.

All authors contributed to shaping data collection and interpretation, drawing on their diverse disciplinary backgrounds and lived experiences. Given the collaborative nature of research in this field, some research participants had pre-existing professional relationships with the research team (CB, CH, KS), as detailed in the COREQ checklist (Appendix A). However, participants had no prior relationship with AJ, who conducted interviews and led analyses, reducing the likelihood that existing relationships influenced analysis. A detailed reflexive statement outlining the author’s positionality and influence on the research is provided in Appendix D and examples of reflexive journalling is provided in Appendix E.

## Demographic Results

### Research experts

We contacted 17 individuals and three professional networks across England, due to their relevance in digital mental health research. Networks included the University of Nottingham’s Institute of Mental Health (IMH), National Institute of Health Research Mental Health Translational Research Collaboration Mission (NIHR MH-TRC), and the Contact, Help, Advice and Information Network (CHAIN). This resulted in 11 individuals expressing an interest in taking part; two had no experience with DMHI trials for CYP, and two were unable to participate due to time constraints. In total, seven researchers participated in the study, all of whom had experiences of being involved in DMHI trials for CYP. Data collection was concluded when interviews no longer appeared to generate novel insights relevant to the research questions [26–28].

The majority were female (n = 5, 71.4%) and located in the UK (n = 6, 85.8%). They were all of White ethnicities, and one disclosed a physical health condition. Researchers worked at different organisations and had different job roles with varying degrees of experience working with CYP and in DMHI research.

### Lived experience experts

We received 24 expressions of interest in joining the LE group. Due to capacity limits, the first 15 individuals to respond were invited to one-to-one meetings via Microsoft Teams. Following these meetings, six young people were selected (17–24 years age range), based on their lived experience with mental health conditions and/or use of DMHIs, as well as ensuring ethnic and LE diversity within the group. All members had experiences of mental health conditions, neurodevelopmental differences, and/or experience with DMHIs.

In the first LE workshop, all six members were present and involved in discussions. In workshops two and three, two members were unable to attend, but one contributed offline.

## Findings

For RQ 1: What is an informative trial? Informativeness was defined across components considered key to informative trials, resulting in the generation of four themes and 10 sub-themes. For RQ2: What contributes to informativeness? Three themes were developed. These themes and five sub-themes reflect activities, abilities and considerations that impact a trial’s informativeness. Themes and subthemes can be viewed in the table below. Following each theme, reflections and insights from the LE workshops are presented to provide additional context, highlight areas of consensus or divergence, and illustrate the lived experience perspective. See Appendix F for our coding tree.

## RQ1: What is an informative trial?

### Theme 1: Addresses questions important to partners

Trials need to answer research questions that are important to key partners, like patients and services, which then result in changes to practice. For example, *“…does it [the trial] answer the questions I want it to answer?”* (R1). This was seen as central to the value of a trial:

*If that's not going to answer the question that we want to answer, then we're wasting a lot of time, money, and resources. So, it's really fundamental that we are informing practice, informing policymakers...because otherwise, how do we enact change?* (R7).

Researchers highlighted that certain research questions may be more important to different people: *“If I'm thinking of informativeness for a commissioner, then, maybe it's more about cost effectiveness….For a young person themselves, they probably want to know, does it work, but also what does it need me to do?”* (R1).

#### Lived experience perspective

LE experts reinforced the idea that research needs to address questions important to different partners. However, they also emphasised that informative research considers its contribution to existing knowledge and communicates impact to patients. They raised concerns surrounding power, where not all partners have equal influence over which questions get prioritised, especially people with lived experience.

### Theme 2: Feasible to conduct

Constructed from research experts’ suggestions that one of the foundations of an informative trial was feasibility, Theme 2 explains how a trial was considered uninformative if it was first and foremost, not deliverable: “*I think that's a good starting point to check for how feasible, you know, a study is to deliver*.” (R7). Three sub-themes were generated on feasibility: 2a) recruitment and retention, 2b) assessment, and 2c) capability.

## Subtheme 2a: Recruitment and retention

Researchers highlighted that feasible trials have *“…the ability to recruit.”* (R4). In line with recruitment capability being seen as a fundamental component of feasibility, another researcher questioned the ability *“…to get the sorts of numbers that you need to sort of demonstrate that it's going to be somewhat viable”* (R8). Challenges recruiting particular populations, such as CYP, were discussed* “…[it] is tough in child and youth mental health…not only do we have to get the young person to assent, we have to get their parent to consent.”* (R8).

Recruitment metrics were a means to understand whether a trial and intervention align with the needs and capacities of both participants and the services delivering them. For example, *“…your retention rate will tell you a lot…if it is something that, you know, a service doesn’t necessarily have capability to embed… they’re not going to recruit to target on time”* (R2).

Retention was seen as an indication of whether an intervention was user-appropriate, highlighting that the intervention itself can impact participant loss:

*…no one in the real world is going to sit there for two hours on [an intervention] …this is where research attrition comes in… you’ve not thought about the end-user.* (R7).

This was also considered essential to trial integrity, with concerns about how participant attrition impacts data quality: *“To retain participants so they complete all the follow-ups… every missed participant, it's a loss to the trial and we have to think about…how to deal with this missing data.”* (R4).

## Subtheme 2b: Assessment

Researchers also mentioned that feasibility included the ability to collect relevant, high-quality data that provides *“…correct and relevant information to be able to answer a question”* (R1). When discussing whether trials were capable of informativeness, participants highlighted the importance of balancing efficiency and acceptability in collecting data: *“the ability to collect data in a way that is, on the one hand, I guess, efficient for the research team and, on the other hand, acceptable to people who take part…”* (R4).

## Subtheme 2c: Capability in feasibility

Researchers emphasised that a key aspect of feasibility is whether the trial can be delivered within the constraints of existing capability, including time, staffing, and broader system capacity: *“… does the site have the time, the resources, the staff to deliver all of this information and collect that data?”* (R7).

Time was critical and often underestimated in how it impacts the feasibility of a trial:

*Have they [healthcare staff] got the time? I think that's critical, because even just asking them to have a brief conversation with a young person about recruitment, you're perhaps asking them to add in something to a workload that they're already too busy [for].* (R1).

Researchers also emphasised the need to identify and plan for what is required to deliver a trial: *“…even a well-designed trial can work out to be not feasible if the infrastructure in the sites isn’t there…”* (R7).

## Lived experience perspective

The LE members agreed that feasibility was an important component of informativeness and was related to trials being easy to understand, safe, and realistic in line with the time and resource available. They highlighted concerns surrounding Theme 2 since they believed it places a lot of emphasis on logistics. They suggested that conceptualisations of feasibility typically don’t place enough weight on the feasibility of the trial to the end-user, suggesting that “feasible to conduct” may not be the same as “feasible to participate in”. In response, they wished to add that for a trial to be feasible, it needs to be realistic for the end-user to use and understand.

### Theme 3: Trustworthy and credible

Researchers consistently linked trustworthiness to informativeness: *“…it comes back to the integrity side of things.”* (R7), suggesting that we should be looking *“…at the credibility”* (R2) of research. Areas of trustworthiness were identified across domains, leading to the development of four sub-themes: 3a) designing trials, 3b) designing interventions, 3c) designing outcomes, and 3 d) transparent reporting.

## Subtheme 3a: Designing trials

Researchers highlighted that trials need to be designed rigorously to inform relevant change-makers: *“How are we going to convince stakeholders to make changes about that if we don't deliver quality, well-designed, impactful research?”* (R7). They emphasised that an informative trial should be *“… properly powered”* (R4), emphasising the importance of credible evidence generation. Alternatively, researchers from clinical backgrounds reflected that this emphasis can make research less reflective of real-world use:

*Some trials do a lot to ensure they're really rigorous from a research point of view, it can actually then mean that the procedures don't really look like what they will in the real world... Have we added things that then mean that it's not so informative because actually it's telling us how it works when it's in a highly controlled, rigorous research world and not in a real world?* (R1).

Researchers highlighted the importance of the trial design matching the needs of the target audience. This underscores the value of lived experience in shaping trial design: *“If we are informed from a lived experience, then that helps us to create a well-designed project, a project that would then help the person that it was designed to help.”* (R7).

## Subtheme 3b: Designing interventions

In subtheme 3b, the design of interventions was highlighted as a component of trustworthiness. Trustworthiness in interventions was described as stemming from a rigorous development process, theoretical grounding, and practical relevance. Researchers described the importance of following a structured development process to ensure that interventions were credible and robust: *“Everything needs…to go through the process [of] development, feasibility, you know, evaluation and implementation… [research] follows that path because we have some rigour.”* (R6).

Researchers expressed concerns about the lack of theoretical grounding in some DMHIs: *“Sometimes these digital trials, you don't really know what the theoretical foundation is”* (R3), a particular concern among commercially developed interventions: *“You get this sort of divide between what's out there in the real world, which is usually commercially developed… And there's possibly absolutely no evidence for their effectiveness.”* (R8).

## Subtheme 3c: Designing outcomes

For a trial to be considered trustworthy and informative, researchers emphasised the need for outcomes that are meaningful, relevant, and appropriately timed. This included outcomes exploring clinical efficacy as well as how participants engage with and apply the intervention in real-world contexts. Researchers stressed the importance of selecting *“the right outcome measures”* (R2) that directly reflect the trial’s aims and are capable of answering the research question: *“you need to ensure that whatever you're collecting is actually going to be able to answer the question that you want it to answer”* (R2). Nonetheless, as this participant queried, *“Does it measure it at the right time points?”* (R1), even well-chosen outcomes can lose value if measured at inappropriate timepoints.

## Subtheme 3d: Transparent reporting

Transparent reporting was seen as foundational for informative trials, including providing comprehensive details about the intervention and trial. This level of transparency was seen as critical for understanding what was tested and how it might be used in other settings:

*…we really need to detail things like how, what platform was it? Was that only available on certain devices? How did people get access to it? Did they need to download things? Was there support and at what points? Was that synchronous, was it asynchronous?* (R1).

## Lived experience perspective

LE experts believed trust was integral to all aspects of research when involving end-users. They agreed that theoretical grounding was a concern in some DMHIs, but also expressed concerns about how they could differentiate a digital intervention emerging from research from the many apps that exist online*,* implying doubts about discerning trustworthiness in publicly available DMHIs. In relation to Subtheme 3c, although LE experts understood that standardised outcome measures were important for validation, they questioned whether these measures were always appropriate, suggesting that revisions and adaptations made through working with relevant partners were important.

LE members also underscored the value of evaluating researchers’ intrinsic motivations. Consistent authorship within a field was perceived as indicative of genuine interest, enhancing trust in the research. For example, they highlighted that they’d look at whether the organisations involved have done work in that area before and author’s affiliation*.* In contrast, concerns were raised about research driven primarily by institutional or funding obligations, which was seen as potentially compromising trust.

### Theme 4: Accessible and safe

Researchers highlighted that informative trials need to be *“… accessible to all, its meeting the needs of everybody.”* (R2), but that identifying safety and accessibility is *“…actually really quite difficult to assess”* (R8). This led to the generation of three sub-themes for Theme 4 that highlight considerations for 4a) patient safety, 4b) accessible communication, 4c) reaching the right populations.

## Subtheme 4a: Patient safety

Researchers mentioned that an informative trial must be conducted in a way that safeguards participants and adheres to ethical standards. This includes the systematic monitoring and transparent reporting of *“Any unintended consequences…captured through recording of adverse events”* (R4). They were concerned about how services manage risk in remote interventions involving young people, which can make it difficult to safely implement DMHIs in clinical practice. For example, *“…that's [managing risk] a big part of why I think things don't get out in the real world as well because, you know, there's a lot of concern about how that's managed”*(R8).

## Subtheme 4b: Accessible communication

For a trial to be informative, its findings must be accessible and understandable to a range of key partners, such as clinicians, policymakers, and the public. Researchers highlighted the importance of research being *“Open Access…”* (R1). Dissemination was seen as a process that extends beyond academic journals, *“…not just publishing the article, but actually yeah, reaching a wider audience”* (R3), with researchers suggesting *“You would want to be looking for lay summaries or executive summaries…”* (R7).

## Subtheme 4c: Reaching the right populations

Subtheme 4c identified that an informative trial must clearly report who participated, how they were recruited, and whether the sample reflects the diversity of the population affected by the condition being studied: *“Are we reaching the populations that we think we are?”* (R3). Representation was seen as essential for supporting equitable research: *“One of the things that's missing quite a lot of the time is that demographic information…that is huge in terms of knowing whether that research is equitable or not”* (R7).

## Lived experience perspective

LE agreed that patient safety was integral, as in Subtheme 4a, but also introduced data confidentiality and privacy, saying they would want to know how their personal information is handled. They also suggested that trustworthiness was pre-emptively attached to the concept of safety, suggesting a broader conceptualisation of safety than what was generated from the research perspective. LE experts agreed with the research perspective that collecting demographics to understand reach was important, highlighting that demographic reporting does not always capture the uniqueness and nuances of individuals. The language to which demographics were described was also mentioned, and they emphasised the importance of avoiding blame-oriented language, such as using phrases like ‘hard to reach’.

In contrast to the research perspective, LE experts mentioned challenges in identifying the degree to which risk is appropriate or inappropriate. They said that some degrees of risk may be necessary for mental health improvement. Understanding the degree to which risk was appropriate was difficult, and they suggested that acceptable risk levels should be co-defined with potential end-users.

## RQ2: What contributes to informativeness?

### Theme 5: Partner involvement

Theme 5 highlights the role of partnerships and community involvement. Researchers emphasised that informative trials are built on collaboration with partners, including clinicians, service users, and the broader public. This involvement was described in terms of both co-development and PPI activities.

Involving partners through co-development and/or PPI activities was considered essential: *“It is about co-production…how do you know your design is going to be acceptable if you're not talking to the people that you want to deliver it for?”* (R7). Involvement was seen to enhance methodological rigour and increase feasibility, *“by having that co-production…it's got more chance of actually being able to address its aims”* (R2).

However, researcher participants raised concerns about tokenistic or misaligned involvement practices, such as involving different age groups, or seeking only confirmation of predefined ideas: *“We tend to go into PPI, once we've already got a really key well-designed sort of idea, and we just say what we're looking for is affirmation and confirmation that that's a good design.”* (R7).

#### Lived experience perspective

LE experts agreed that partner involvement was an important contributor to informativeness. However, they also highlighted the importance of other forms of involvement. Emphasis was placed on involving charities and organisations with strong ties to potential end-users, as well as involving less heard groups that don’t usually have ‘leverage’ to take part. They mirrored the concerns of researchers regarding tokenistic PPI practices, discussing the importance of partnerships and community involvement at the earliest stages of research.

### Theme 6: Researcher and site capacity

Researcher participants highlighted that informativeness arises from having individual and site capacity: *“…you’ve got to think about setting, infrastructure, and resources…”* (R7). Three sub-themes were generated in relation to capacity: 6a) skills and expertise, 6b) working together, and 6c) infrastructure.

#### Subtheme 6a: Skills and expertise

Subtheme 6a highlights the need for different skills and expertise when conducting an informative RCT. Skills related to digital, clinical and researcher domains were mentioned by researchers: *“…there's a raft of [needed] research skills.”* (R1). They noted that training and expertise would *“very much depend on…what it is that you’re developing”.* (R2).

Researchers emphasised that digital expertise was required at all stages of DMHI research and development to ensure long-term functionality. Participant R6 said that *“digital interventions need to be maintained and updated”,* suggesting that having “*… programmers on board, you know, that's essential*” when developing DMHIs.

It was considered important to provide training to staff to facilitate consistent and safe trial delivery: *“Do they understand what the participant should and shouldn't be doing? Do they understand about things like protocol deviations?…I think sometimes research breaks down just because of a lack of experienced or well-trained staff.”* (R7).

#### Subtheme 6b: Working together

Researchers described the importance of having a diverse team involved in the trial: *“…you need different people … we have a lot of researchers who are working on facilitating the trial itself. So that's PhD students, master’s students, postdocs, and then you have more experienced senior researchers.”* (R3). Collaboration with specific experts, like commercial partners and methodologists, was seen as essential for developing and sustaining engaging digital interventions: *“…we're also working together with like private companies to deliver the technology.”* (R3).

However, other researchers were sceptical about the motivations and sustainability of commercial involvement: *“I don't see them really working [working with a commercial company] …what does the private entity get out of this?”* (R8), suggesting these collaborations may be misaligned with the goals of improving access to mental health treatment. A disconnect between the priorities of funders and research implementation appeared to contribute to perceptions that evidence-based interventions may be overshadowed by commercially developed products: *“… the funders for research, you know, they're fully committed to endless evaluations. They don't want anything really to do with dissemination…that's someone else's problem.”* (R8).

#### Subtheme 6c: Infrastructure

Researchers described that the informativeness of a trial depends heavily on the availability of adequate infrastructure, such as time, finance and technology, that match both research and clinical settings. They indicated challenges with involving healthcare staff in research when services are already under pressure: *“That willingness [by clinicians] really to promote research…is very hard because with all the pressures that are put on these services”* (R4). Constraints with current infrastructure availability were seen as a potential barrier to delivering the research both during and post-trial: *“IT services [in the NHS] tend to be quite, you know, they're in-house, and they have their own way of working, and they don't always reflect, you know, out the outside world…”* (R6).

#### Lived experience perspective

LE experts agreed that researcher and site capacity contributed to informativeness. When discussing Subtheme 6a, they suggested that researchers have additional ‘soft’ skills, e.g., empathy, listening and communication skills. LE experts also considered the capacity of end-users, with the belief that supporting participant involvement (e.g., with technical support) promoted trial informativeness.

### Theme 7: The wider context

Theme 7 considers the wider context and its relationship with trials: *“It's looking at everything, so the bigger picture, and then for me that would be, what would inform the delivery of a successful trial.”* (R7). There were concerns about whether research ever reaches clinical application, ultimately leading to research waste: *“…we get like, pilotitis, which is what I think the whole field is stuck in, so interventions never get to the real world because we're just constantly doing evaluations.”* (R8). Two sub-themes were generated that discuss specific considerations within 7a) clinical environments and 7b) research environments.

#### Subtheme 7a: Clinical environments

Researchers indicated that trials must be designed with the realities of clinical environments and service pressures, including the potential role of DMHIs in addressing gaps in care: *“So in terms of mental health treatment, for example, it's a chronically underfunded. So how are we going to make that better for the end user?”* (R7). A trial being embedded in the intended setting was considered important: *“We have some trials that are within the sort of child and adolescent mental healthcare services…I think that's also a lot more informative than when we have these trials running outside that context.”* (R3). However, researchers mentioned that* “…primary care would be incredibly difficult to deliver.”* (R7)*,* signifying that we need to consider the appropriateness of a clinical setting for trial delivery. As a result, researchers highlighted the importance of designing interventions that “*can seamlessly fit into…their environment”* (R2).

#### Subtheme 7b: Research environments

Researchers identified several broader challenges within the research environment, seen as barriers which impacted all components of informativeness. This included *“positive bias in reporting”* (R7), with it being *“…difficult to publish those results”* that don’t show effectiveness (R8). A lack of reporting on *“how the intervention was actually developed”* (R7) was also noted.

Trial registrations to identify trials that were not published was suggested:

*When they start, you need to register it and so on. And I'm just thinking about these trials that...did not recruit the sufficient number because I guess you won't get it from a published paper, because there is no paper.* (R4).

Researchers highlighted concerns with the mismatch between slow-paced traditional trial procedures and the fast-changing landscape of digital technologies: *“The technical capability of, um, devices and, and software, you know, can be surpassed by this sort of long [research] cycle.”* (R6).

#### Lived experience perspective

Discussions throughout our workshops coincided with elements of this theme, with LE members emphasising the importance of appropriateness to end-users. Some members felt that digital mental health interventions may not benefit everyone, believing that some people do prefer face-to-face contact. This coincided with subtheme 7a, that not everyone will benefit from every therapeutic tool.

## Discussion

**Fig. 2:**
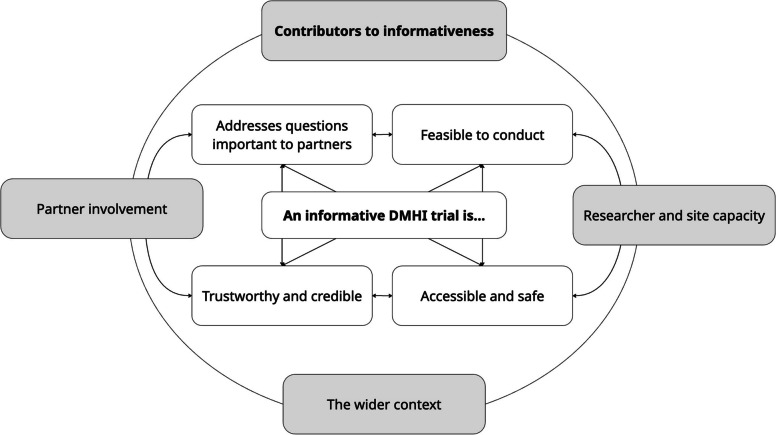
Themes and relationships between themes

With challenges with uninformative studies [2–4, 10], and the lack of guidelines for informative trials in the context of digital interventions for CYP mental health, this qualitative study aimed to explore the perspectives of researchers and young people on what constitutes an informative trial. Our study offers specific context-relevant guidelines tailored to DMHI trials for CYP. See Fig. 2 for an overview of themes and their relatedness.

### Principal findings

The findings indicate that informativeness is a multi-faceted concept and those involved in the design, delivery and reporting of trials should consider multiple strategies to improve the usefulness of their trials. Each theme identified a distinct aspect relating to informativeness; nonetheless, each aspect interconnects and reinforces others. For instance, Theme 1: Addresses questions important to partners and Theme 3: Trustworthy and credible, both rely on partner engagement. However, Theme 1 steers research toward partner priorities, whereas Theme 3 safeguards methodological rigour. Likewise, the drivers of informativeness (Themes 5–7) directly reinforce its foundational components (Themes 1–4). Several themes appear specific to the DMHIs or CYP; for instance, recruitment challenges related to acquiring both parental consent and assent (Subtheme 2a), concerns of commercially developed digital treatments lacking evidence (Subtheme 3b), and the type of capacity required, such as digital expertise (Theme 6). However, not all themes generated are unique to this context, and many are addressed within existing regulations [29]. This study therefore provides insight into how these are interpreted, prioritised and negotiated when conducting DMHI trials for CYP.

Similarities and differences were noted between researchers and LE experts. Researchers placed more emphasis on the logistics, rigour, and methodology of trials, while LE experts highlighted broader issues concerning power dynamics, data protection, and discerning the trustworthiness of science. Despite these differences, both perspectives offer valuable strategies for strengthening the informativeness of trials. Including both perspectives enabled a richer, more nuanced perspective of informativeness, guiding the design of trials that are not only methodologically rigorous but also relevant, acceptable and meaningful to the young people they aim to benefit.

In relation to the current literature, these findings extend the work of Zarin et al. [5], highlighting that informative trials ought to consider components beyond trial importance, design, feasibility, integrity and reporting. The findings generated from this study also suggest that the fulfilment of these components can be identified in numerous ways*.* For example, it has been proposed that feasibility is demonstrated through reaching recruitment targets [4, 5]; however, as we show, it can also be indicated through retention, assessment and resource metrics. Safety and accessibility were highlighted as components of informative trials, which coincided with O’Dea and colleagues’ [10] study, where ethical and equitable research was considered important. We also identified divergences in perspectives between LE and research experts, further supporting the challenges O’Dea faced in collating priorities across groups.

Involving relevant partners in the planning and delivery of research was seen as a contributor to informativeness, supporting the findings of Prowse and colleagues [7] in their scoping review. Existing frameworks on informativeness do not consider involving partners [5, 9, 10], despite the knowledge that partner involvement improves the quality of research [30, 31] and is recommended in trial guidelines [29]. Since it has potential to support informativeness, adaptations to these frameworks are needed. Another novel finding is that the capacity of researchers and sites was seen as a contributor to informativeness. While Taylor & Kowalkowski [8] suggest that identifying capacity in pilot studies may improve the informativeness of subsequent trials, no other study has considered capacity when conceptualising informativeness. Studies would benefit from identifying and building capacity prior to trial conduct to support informativeness.

### Strengths, limitations and future directions

This study acknowledges limitations that should be considered when interpreting the findings. This includes the reliance on self-reported data and the potential for selection bias in both the LE and research expert samples. The sample size was small and purposively selected, meaning it was neither representative nor exhaustive. Our LE group were ethnically diverse, but the majority reported prior experience as a PPI contributor or research participant, suggesting a level of engagement with research that is not typical of the wider youth population. While this experience enriched their contributions, it may also mean their perspectives differ from those of young people less involved in research. Our research expert participants were predominantly white and female, a common profile in research studies that limits the range of perspectives captured. Future work may benefit from including diverse communities to identify whether informativeness guidelines are inclusive and culturally sensitive. While the inclusion of two partner groups added valuable perspectives, other relevant voices, such as funders and policy makers, were not included and may offer additional insights not identified here. The interviews with researchers were relatively brief to support participation; however, this limited opportunities to explore complex ideas in depth. Lastly, the study was situated within the context of DMHI trials for CYP, but the findings do not exclusively highlight components specific to youth DMHI trials. Several themes reflect broader issues applicable across clinical trials and age-groups. As such, the findings should be interpreted as contextualising general trial considerations within a youth DMHI setting.

Despite these limitations, the study offers several important strengths. To our knowledge, this is the first study to explore how LE young people conceptualise informativeness. By including both researchers and LE experts, the study embraced diverse perspectives, recognising that informativeness is not only shaped by scientific considerations, but also by values, priorities and lived experience. As the first qualitative study to explore informativeness alongside LE voices, it allowed for a better understanding to emerge than would be possible through theoretical or quantitative approaches. The use of both semi-structured interviews and participatory workshops provided methodological triangulation, enhancing the trustworthiness of findings. Importantly, the design reflects a reflexive and participatory research ethos, aligning with the broader shift towards co-produced knowledge in health research. It also demonstrates that involving non-academic contributors in shaping methodological concepts is viable and valuable.

This study provides a foundation for future work in several areas. Future work should incorporate these findings into the design and evaluation of trials, particularly through further involvement processes. It would be valuable to involve a wider range of partners, such as policymakers, through mixed-method approaches for a broader understanding of the challenges and opportunities in delivering and engaging with DMHI trials. This would further our understanding of what makes a trial informative in practice and how different partners interpret and prioritise informativeness within trial contexts.

Another next step would be to conduct a systematic review of the informativeness of existing DMHI trials for CYP. Such a review could assess how often key features of informativeness identified here are reported and addressed, helping to establish a baseline and identify common gaps in the current evidence base. However, before a review can take place, there is a need to develop or refine existing frameworks and guidelines for assessing trial informativeness. These frameworks should be conceived as evolving, working documents that can adapt to the rapidly changing landscape of research, societal priorities and emerging technologies.

## Conclusion

This qualitative study explored the perspectives of researchers and young people on what an informative DMHI trial is for CYP. We generated four themes and 10 sub-themes addressing RQ1 (“What is an informative trial?), and three themes with five sub-themes for RQ2 (“What contributes to informativeness?”). Each theme captures a distinct domain of informativeness, yet together they weave into a thematic map of drivers (Themes 5–7) that reinforce core pillars (Themes 1–4). These findings guide researchers to enhance the clinical utility of DMHI trials. Importantly, this study is among the first to demonstrate the possibility and value of involving lived experience contributors in shaping informativeness. While the study was limited by the range of participants included, it underscores the importance of engaging a broad set of partners with diverse experiences to deepen understanding. Future research should assess the current informativeness of DMHI trials for CYP and develop strategies to strengthen their impact. Together, these insights move the field closer to ensuring that trials are not only methodologically sound, but also meaningful to those they intend to serve.

## Supplementary Information

Additional file 1: COREQ Checklist (Appendix A), and GRIPP2 checklist (short-form) (Appendix B).

Additional file 2: Topic schedule (Appendix C).

Additional file 3: Reflexive statement (Appendix D), and examples of reflexive journaling practices (Appendix E).

Additional file 4: Coding tree (Appendix F).

## Data Availability

The full dataset, including interview transcripts, is not available due to ethical and privacy restrictions. Participants provided consent for the publication of anonymous quotes only.

## Abbreviations

ADHD: Attention-deficit hyperactivity disorder
CYP: Children and young people
DMHI: Digital mental health interventions
LE: Lived experience
NIHR: National Institute of Health Research
PTSD: Post-traumatic stress disorder
RCT: Randomised controlled trials
UK: United Kingdom
