# Supplementary material for: Understanding trial informativeness in digital mental health: perspectives from researchers and lived experience experts
Source: Trials. 2026 Mar 18;27:322. doi: 10.1186/s13063-026-09610-w (PMC13112908; doi:10.1186/s13063-026-09610-w)
Supplement: Supplementary file 1 — Additional file 1: COREQ Checklist (Appendix A), and GRIPP2 checklist (short-form) (Appendix B). [file 13063_2026_9610_MOESM1_ESM.docx]

Appendix A: The consolidated criteria for reporting qualitative research (COREQ)

| Domain 1: Research team and reflexivity | **Further details** | | **Location in manuscript** | |
| --- | --- | --- | --- | --- |
| Personal Characteristics | | | | |
| 1. Interviewer/facilitator Which author/s conducted the interview or focus group? | AJ – interviews and workshops  CB – workshops  SO – workshops | | p.7 | |
| 2. Credentials  What were the researcher’s credentials? E.g. PhD, MD | AJ – Msci  CB - PhD  KS - PhD  CLH - PhD | | See reflexive statement. | |
| 3. Occupation  What was their occupation at the time of the study? | AJ – PhD student  CB – Research Fellow  KS – Assistant Professor  CLH - Principal Research Fellow | | See reflexive statement. | |
| 4. Gender Was the researcher male or female? | Four females | | See reflexive statement. | |
| 5. Experience and training  What experience or training did the researcher have? | CB, CH, KS have previous experience facilitating and publishing research using interview and qualitative methods, and extensive experience in clinical trials and mental health research.  AJ has some experience of conducting interviews and has received training in qualitative methods, as well as their application clinical trials. | | See reflexive statement. | |
| Relationship with participants | | | | |
| 6. Relationship established  Was a relationship established prior to study commencement? | Mixed. Some of the research participants had established relationships to the authors (CB, CH, KS) but not AJ. These were unavoidable due to the collaborative nature of this research field. However, since AJ led the data collection and analysis, it was unlikely that this impacted study findings. Other participants had no relationship to the authors. | | p.9 | |
| 7. Participant knowledge of the interviewer  What did the participants know about the researcher? e.g. personal goals, reasons for doing the research | All participants were briefed on the purpose and aim of the study which was to explore their perspectives on trial capability. Ethical approval was obtained for the study. All researcher participants provided informed consent before taking part in interviews. | | p.6, p.43 | |
| 8. Interviewer characteristics What characteristics were reported about the interviewer/facilitator? e.g. Bias, assumptions, reasons and interests in the research topic | Since CB, CH, KS have experience in mental health research, this might have been a source of bias. However, they were not involved in conducting interviews, so it is unlikely this influenced data collection. | | See reflexive statement. | |
| Domain 2: study design | | | | |
| Theoretical framework | | | | |
| 9. Methodological orientation and Theory  What methodological orientation was stated to underpin the study? e.g. grounded theory, discourse analysis, ethnography, phenomenology, content analysis | A qualitative multi-method design, guided by pragmatism. This involved interviews and workshops.  Interviews were analysed using reflexive thematic analysis. Workshops were not formally analysed but are described in paraphrased form in relation to thematic data. | | p.5-9 | |
| Participant selection | | | | |
| 10. Sampling  How were participants selected? e.g. | Researchers were purposively and snowball-sampled via email and word-of-mouth.  Lived experience experts were conveniently recruited through the McPin Foundation. | | p.5-6 | |
| 11. Method of approach How were participants approached? e.g. face-to-face, telephone, mail, email | Email | | p.6 | |
| 12. Sample size How many participants were in the study? | Seven research participants and six lived experience experts. | | p.10-11 | |
| 13. Non-participation How many people refused to participate or dropped out? Reasons? | No participant withdrew consent or dropped out. A total of 11 individuals expressed an interest in taking part. Of these, two had no experience with DMHI trials for CYP, and two were unable to participate due to time constraints. | | p.10 | |
| Setting | | | | |
| 14. Setting of data collection  Where was the data collected? e.g. home, clinic, workplace | | Data was collected via Microsoft Teams. | | p.7 |
| 15. Presence of non-participants  Was anyone else present besides the participants and researchers? | | No | | NA |
| 16. Description of sample  What are the important characteristics of the sample? e.g. demographic data, date | | These are described in-depth in the paper. | | p.10-13 |
| Data collection | | | | |
| 17. Interview guide Were questions, prompts, guides provided by the authors? Was it pilot tested? | | We used a topic guide to facilitate interview discussions. This was pilot tested with an individual outside of the research team. | | p.7 |
| 18. Repeat interviews  Were repeat interviews carried out? If yes, how many? | | No | | NA |
| 19. Audio/visual recording  Did the research use audio or visual recording to collect the data? | | All research interviews were video recorded via Microsoft Teams. Workshops were not recorded. | | p.7 |
| 20. Field notes  Were field notes made during and/or after the interview or focus group? | | Field notes were taken during and within 24 hours of conducting interviews by the researcher involved in data collection (AJ). | | p.9-10 and reflexive statement. |
| 21. Duration What was the duration of the interviews or focus group? | | Approximately 30 minutes. | | p.7 |
| 22. Data saturation  Was data saturation discussed? | | We were restricted by available resources to conduct many interviews. However, saturation was discussed during data collection and was not deemed an issue by the research team. An additional interview was conducted, where we felt that no new themes were being generated. | | p.10 |
| 23. Transcripts returned Were transcripts returned to participants for comment and/or correction? | | No. | | NA |
| Domain 3: analysis and findings | | | | |
| Data analysis | | | | |
| 24. Number of data coders  How many data coders coded the data? | | One. | | p.8 |
| 25. Description of the coding tree  Did authors provide a description of the coding tree? | | See appendices | | p.14 and appendices. |
| 26. Derivation of themes  Were themes identified in advance or derived from the data? | | Themes will be derived from the data and an understanding of the literature surrounding trial capability. | | p.8 |
| 27. Software What software, if applicable, was used to manage the data? | | NVivo15 Pro. | | p.8 |
| 28. Participant checking  Did participants provide feedback on the findings? | | No. | |  |
| Reporting | | | | |
| 29. Quotations presented Were participant quotations presented to illustrate the themes / findings? Was each quotation identified? e.g. participant number | | Themes and sub-themes are supported with direct quotes attributed to anonymised participants by their role (R=Researcher) and a unique number. Responses from lived experience experts are described in paraphrased form. | | p.8-9 |
| 30. Data and findings consistent  Was there consistency between the data presented and the findings? | | Yes. | | p.14-29 |
| 31. Clarity of major themes  Were major themes clearly presented in the findings? | | Yes, key themes are described in relation to research question one and two. | | p.14-29 |
| 32. Clarity of minor themes  Is there a description of diverse cases or discussion of minor themes? | | Yes, sub-themes are described in relation to the research questions. | | p.14-29 |

Appendix B: GRIPP2 Checklist

| **Section and topic** | **Item** | **Reported on page no.** |
| --- | --- | --- |
| 1: Aim | Report the aim of the study | p.4 |
| 2: Methods | Provide a clear description of the methods used for PPI in the study | p.5-10  PPI described as lived experience experts. |
| 3: Study results | Outcomes—Report the results of PPI in the study, including both positive and negative outcomes | p.12-29 |
| 4: Discussion and conclusions | Outcomes—Comment on the extent to which PPI influenced the study overall. Describe positive and negative effects | p.30 |
| 5: Reflections/critical perspective | Comment critically on the study, reflecting on the things that went well and those that did not, so others can learn from this experience | p.30-33 |
