# Supplementary material for: Understanding trial informativeness in digital mental health: perspectives from researchers and lived experience experts
Source: Trials. 2026 Mar 18;27:322. doi: 10.1186/s13063-026-09610-w (PMC13112908; doi:10.1186/s13063-026-09610-w)
Supplement: Supplementary file 2 — Additional file 2: Topic schedule (Appendix C). [file 13063_2026_9610_MOESM2_ESM.docx]

Appendix C: Topic Schedule

| **Question example** | **Prompts** |
| --- | --- |
| How do you define informativeness? | Ability to guide decision-making? |
| What makes a DMH trial for CYP informative? | Key elements/components.  Does this differ in DMHIs? |
| How do measure/assess the informativeness of a DMH trial? | Any frameworks or guidelines you are aware of?  Challenges in assessing?  How would you explore?  What evidence indicates a trial being highly informative?  What information is needed/important? |
| What would you recommend to improve informativeness? | Are there specific policies, practices, or collaborations you’d prioritise? |
| Is there anything else important that we haven’t covered? | Recommendations, future development in the field? |
