# Supplementary material for: Understanding trial informativeness in digital mental health: perspectives from researchers and lived experience experts
Source: Trials. 2026 Mar 18;27:322. doi: 10.1186/s13063-026-09610-w (PMC13112908; doi:10.1186/s13063-026-09610-w)
Supplement: Supplementary file 3 — Additional file 3: Reflexive statement (Appendix D), and examples of reflexive journaling practices (Appendix E). [file 13063_2026_9610_MOESM3_ESM.docx]

Appendix D: Reflexive Statement

I am a young white female post-graduate student in Applied Psychology, and the primary data collector and analyst for this study. I have an undergraduate degree in Psychology and have no prior involvement in DMHI trials for CYP. This was the first time I had recruited, collected, and analysed data from research professionals.

Having no knowledge of this field had some advantages. For example, it enabled me to approach the data without preconceptions about normal practices in the field, allowing me to be more open to participants’ accounts. On the other hand, it presented some challenges. For instance, I sometimes felt uncertain about how best to engage with participants who had greater domain-specific expertise. In response, I adopted proactive strategies to build rapport, including familiarising myself with participants’ work in advance and expressing genuine interest during interviews. These strategies enhanced the quality of interview discussions. I was also supported by a highly experienced supervisory team who have extensive experience in DMHI trials for CYP and facilitating and publishing qualitative research (CB, KS, CLH). Working with my supervisors, the co-authors of this study, I was able to navigate some of the challenges that arose during interviews and improve my approach. For example, when we noticed that participants weren’t always comfortable defining certain terms, we adapted the questions to be more accessible. This collaboration helped enrich the analytic process and contributed to a more nuanced understanding of the data.

Appendix E: Reflexive Journaling Practices Examples

During the course of conducting interviews, it became evident that the concepts of informativeness central to the study, was not uniformly understood or readily articulated by some participants. While the topic guide initially assumed a shared conceptual understanding, several participants expressed uncertainty or discomfort in defining informativeness without additional context. This prompted a critical reflection on my own initial assumptions surrounding these concepts, and the need for greater flexibility in the interview process. In response, I adapted my approach by offering a brief, neutral description of this concept and subsequently inviting participants to evaluate it’s relevance and applicability to DMHI trials involving CYP.

Upon listening back to recordings and reviewing transcripts, I identified missed opportunities where I could have probed more deeply into participants’ perspectives. For instance, some early participants described PPI as a key component of informative trials, but I did not explore in detail how effective PPI could be delivered or what challenges might be involved. Reflecting on these missed opportunities informed my approach in subsequent interviews, prompting me to revisit these areas with later participants to gain a deeper understanding of their views.
