# Supplementary figures and images for: Understanding trial informativeness in digital mental health: perspectives from researchers and lived experience experts
Source: Trials. 2026 Mar 18;27:322. doi: 10.1186/s13063-026-09610-w (PMC13112908; doi:10.1186/s13063-026-09610-w)

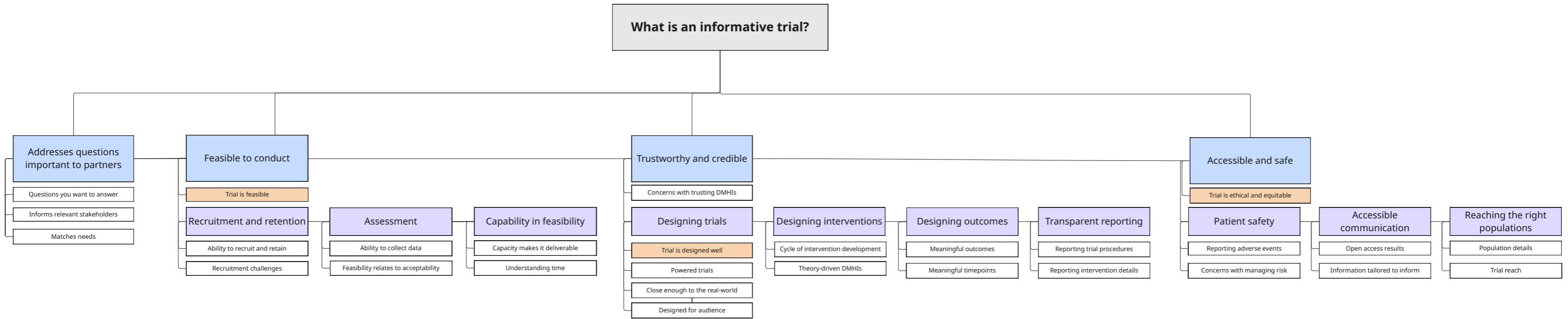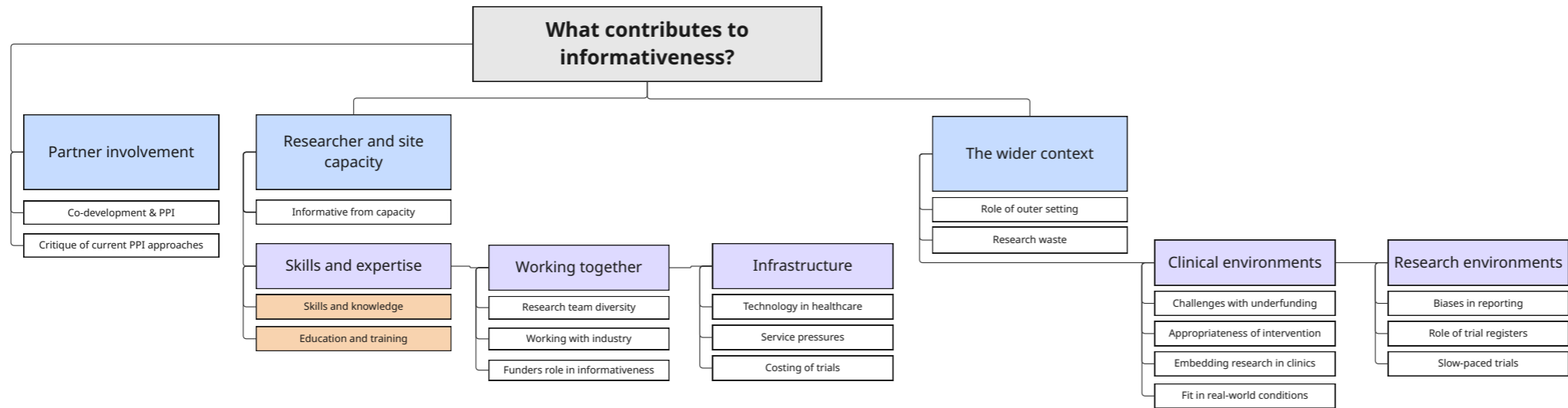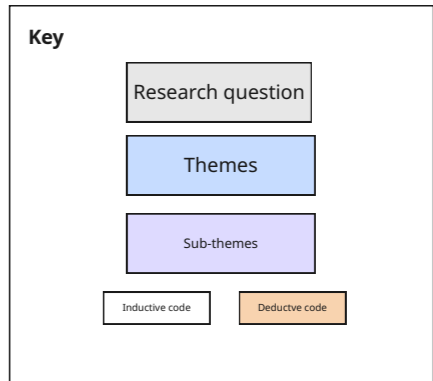

Supplement: Supplementary file 4 — Additional file 4: Coding tree (Appendix F). [file 13063_2026_9610_MOESM4_ESM.pdf]
